# Fears and barriers: problems in breast cancer diagnosis and treatment in Pakistan

**DOI:** 10.1186/s12905-021-01293-6

**Published:** 2021-04-14

**Authors:** Sidra Saeed, Muhammad Asim, Malik Muhammad Sohail

**Affiliations:** 1 Department of Sociology and Social Research, University of Trento, Trento, Italy; 2 Department of Community Health Sciences, Aga Khan University, Stadium Road, PO Box 3500, Karachi, 74800 Pakistan; 3 Department of Sociology, Institute of Arts and Sciences, Government College University Faisalabad, Chiniot Campus, Chiniot, Pakistan

**Keywords:** Breast cancer, Feminine sensitivity, Stigmatization, Inadequate financial resources, Social support, Pakistan

## Abstract

**Background:**

Women in Pakistan lack appropriate awareness about diagnosis and treatment for breast cancer due to a range of multifaceted barriers. There is a dearth of literature examining the socio-cultural factors that inhibit women from breast cancer screening, diagnosis and treatment in Punjab, Pakistan. Addressing this gap, this qualitative study sought to identify and explore the barriers that hinder women from seeking timely screening and treatment.

**Methods:**

In this process 45 women (age = 18–50 years) with breast cancer were purposively sampled and interviewed from the Punjab Institute of Nuclear Medicine (PINUM) hospital, Faisalabad, Pakistan.

**Results:**

An inductive approach was used to analyze the data which resulted in the emergence of eight subthemes under the umbrella of three major themes that delineate individual, socio-cultural and structural barriers to seek screening and treatment of breast cancer in Punjab. Individual barriers included lack of awareness, hesitance in accepting social support, and spiritual healing. The identified socio-cultural factors included feminine sensitivity, stigmatization, and aversion to male doctors. Lack of financial resources and apathetic medical services were structural barriers that hinder screening and treatment.

**Conclusions:**

These barriers can be addressed through raising awareness and community mobilization about breast-self exam and treatment. The healthcare system should also pay attention to socio-psychological and cultural factors impeding women's access to available health facilities.

**Supplementary Information:**

The online version contains supplementary material available at 10.1186/s12905-021-01293-6.

## Background

Breast cancer is the most frequently diagnosed cancer among women worldwide and is the most common cancer, falling only second to lung cancer [1]. The incidence rate of breast cancer is higher in Western European countries as compared to Eastern Asian or African countries [2]. However, survival rates are much higher in Western European countries as compared to low and middle income countries [3]. This is attributed to the significantly better screening and treatment facilities prevalent in high-income countries. In addition, higher awareness about the timely screening of mammography in high income countries has reduced breast cancer mortality in the previous four decades [2, 4–6].

Pakistan has the highest rate of breast cancer in Asia [7] and latest demographic trends suggest that this rate is likely to further increase in the coming years [8]. Diagnosis of breast cancer at an early stage has a significant impact on reducing both morbidity and mortality. Mammographic screening is also linked with a variety of socio-cultural and economic factors [6, 9, 10]. However, women in Pakistan tend to approach health facilities at the last stage of cancer due to plethora of socio-economic and cultural factors such as: age, employment status, lack of awareness, fear of surgery, and belief in traditional treatments, and spiritual healing [11]. In Pakistan, 89% of breast cancer patients are diagnosed at later stage and 59% at an advanced stage due to lack of awareness [12]. The fear of stigmatization and feminine sensitivity limits the choice of treatment and early detection of breast cancer in low and middle income countries [13–16]. In addition, physical barriers also become a source of psychosocial stress, as patients show hesitancy to undergo the exhausting screening and treatment process [17]. In the context of Pakistan, only a handful studies have explored the reasons behind delay in cancer screening and treatment such as lack of awareness [18], poor socioeconomic status [12], availability and affordability of cancer medicine [19], and risk factors (exposure to hazardous industrial substances) [20, 21].

There is a dearth of studies on the impact of socio-cultural beliefs and their impact on delayed breast cancer screening and treatment in Pakistan. A few studies have investigated the patient’s knowledge about breast cancer in Punjab [12, 18], effects of social support in adjustments with breast cancer [22], handling and managing of breast cancer [22] and delayed presentation of breast cancer [12, 23]. Furthermore, Banning et al. (2010) conducted a qualitative study but merely emphasized on cultural context of experience of breast cancer [24]. In Contrast, present study has used qualitative method to explore the fears and barriers of diagnosis and treatment of breast cancer. A unique feature of this study is to simultaneously unearth individual, sociocultural and structural barriers that prevent women to seek timely diagnosis and treatment of breast cancer in Punjab. It has explored the dimensions of breast cancer experience that are scant in existing literature.

## Material and methods

### Study design and setting

This study used a descriptive exploratory qualitative research design to explore fears and barriers associated with breast cancer diagnosis and treatment in Punjab. Such design helps to explore in-depth information about the perceptions, thoughts and behaviors of respondents. For this purpose in-depth interviews were conducted with women who were suffering from breast cancer to unearth fears and barriers for diagnosis and treatment of breast cancer. The study was conducted in Punjab, the largest province of Pakistan where more than 70% people speak Punjabi language. Punjabis are largest ethnic group in country with more than 45% of total population of 220 million. At present, there are seven hospitals exclusively treating cancer patients in Punjab. For present study, the Punjab Institute of Nuclear Medicine (PINUM) hospital Faisalabad was purposively selected due to convenient access to patients there. This hospital covers a population of 14 million across five nearest districts. Permission to collect data from hospital was obtained through a formal application along with research proposal to Director of PINUM. Director designated a female physician from the hospital who introduced researchers with potential study participants which helped to build rapport.

### Study participants

Eligible study participants were breast cancer patients who were aged between 18 to 50 years and were receiving treatment in the hospital. The socio-demographic characteristics (Age, type of residence, marital status, monthly household income in rupees, educational level, and diagnosis history) of study participants are mentioned in Table 1. This study was conducted on purposively selected women (*n* = 45) who were suffering from breast cancer. Three participants refused to further participate in data collection after starting interview due to lack of interest to respond the questions. These interviews were excluded from the completed interviews. Sample size was determined through ‘saturation principle’. When the researchers observed that no new codes and themes are emerging, the further interviews were ceased by the mutual understanding of co-authors. Patients who were visiting the hospital for screening were excluded after the discussion with the concerned physician. Researchers only interviewed the patients who were getting the treatment from the hospital. Inclusion criteria for the respondents were women age 18–50 years, diagnosed with breast cancer with stage 1 and 2 and patients who agreed to participate voluntary in this study. Furthermore, we only interviewed the patients that were able to communicate and respond the questions affectively. Patients at stage 3 or 4 of the disease, patients under any psychological treatment, hospitalized patients and those who declined to voluntarily participate in the study were excluded.

**Table 1:** Socio-demographic characteristics of study participants (n = 45)

Variables	Frequency	Percentage
Age range in years (19–50 years; mean age 35.4 years)		
19–23	06	13.3
24–28	06	13.3
29–33	05	11.1
34–38	10	22.2
39–43	09	20.0
44–48	07	15.6
49–53	02	4.5
Type of residence		
Rural	22	48.9
Urban	23	51.1
Marital status		
Married	30	66.7
Single	10	22.2
Widow/divorced	05	11.1
Monthly income in Pakistan rupees		
Up to 30 thousands	12	26.7
31–60 thousands	21	46.7
61 thousands and above	12	26.6
Education		
Up to middle (8th grade)	05	11.1
Matric to Graduation	33	73.3
Masters and above	07	15.6
Diagnosis history		
Up to 1.5 years	10	22.2
1.6–3.5 years	28	62.2
3.6 years and above	07	15.6

### Data collection

The data were collected through a semi-structured interview guide which aimed to explore the potential barriers to diagnosis and medical treatment of breast cancer. The interview guide was developed by the authors after literature review keeping in view the research objectives. Interview guide was validated through expert review by medical specialists (02), medical sociologists (03) and a social psychologist (01). Interview guide was pilot tested on five patients before data collection. Furthermore, the semi-structured interview was also periodically updated as researchers learned more from respondents about their experiences. Semi-structured interview guide developed for this study is provided as Additional file 1. Two female research assistants were hired and trained about note taking and analysis keeping in view the cultural sensitivity of the research.

To collect the data, patients were physically approached by the research assistants whilst seated in the waiting rooms. After the assessment of eligibility and taking consent, interviews were conducted by the first author (SS) in a separate room within hospital that was designated for conducting interviews in a safe environment. SS had four years’ experience of qualitative data collection prior to this study. During data collection, the hospital staff and family members of participants were not present at the time of interview. Interviews were recorded through a digital device and noted with the consent of the respondents. The first author moderated the session along with note takers. On completion of each interview, a debriefing session between moderator and note takers was conducted to reflect on responses of participant. Each participant was interviewed two times. Researchers did not establish any formal relationship with study participants. However, in first session, participants were briefed about objectives of the study, need of study, process of interview and their consent was acquired.

In second session, interviews were recorded. Each interview took 14 to 47 min (mean = 29 min). Total interview time of 45 participants was 1337 min. Interviews were conducted in Punjabi language. Data collection took place from 22^nd^ January 2018 to 30^th^ April 2018.

### Data analysis

**Fig. 1:**
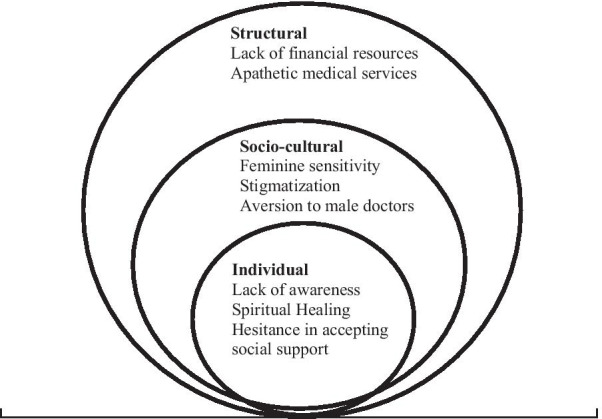
Socioecological model highlighting fears and barriers in breast cancer diagnosis and treatment in this study

Data of this study were analyzed through the inductive method [25]. All these recorded interviews were transcribed verbatim by the SS and MMS from Punjabi to English. MA and SS read transcripts and wrote notes to ensure the originality of the data. The analysis approach used a combination of predetermined and self-derived themes facilitated by field observation, discussions with patients, attendants and doctors. Furthermore, the themes and sub-themes were identified by all co-authors (MMS, MA) and research assistants during a detailed reading of the transcripts, recordings and field notes. A codebook was generated from the data as well as concepts of interest at the outset of the study that was periodically reviewed by all co-authors. Each time after reading transcripts, the emerged codes, categories and themes were identified and written on a structured codebook. MA and SS discussed mutually the themes and sub-themes of the study by reviewing the quotes of the codebook. The study themes, sub-themes, and quotes were discussed among all the co-authors and discrepancies were discussed during the analysis and interpretation of the data. The study themes were organized into three broad categories i.e., individual, socio-cultural and structural level barriers. The themes and sub-themes are presented in socioecological model (Fig. 1).

### Ethical consideration

This study was approved by the ethical review board and advance research board (GCUF/ARB/09/2017) of the Government College University Faisalabad, Pakistan. The written consent to interview the patients was also obtained from the administration of the PINUM hospital. Before data collection, written and oral consent of respondents was taken and prior to the interviews the objectives of the study were briefed to the respondents. The anonymity and confidentiality of the participants were ensured during data collection and reporting of the study findings.

### The researchers’ reflexivity

In qualitative research, reflections are mostly based on interactional setting in which interviewer and respondent conduct the interview from different perspectives, with different expectations and objectives, having different social roles, theoretical backgrounds, and positions [26]. These differences certainly influence the interaction in the interview situation making researcher’s positionality pivotal in qualitative research. In the current study, interviewers were not members of the hospital staff. When interviewers entered the interview situation, they were not considered outsiders because hospital staff introduced interviewers to respondents. Respondents considered them to be a part of hospital staff who are interested to learn from the patients about their experiences of diagnosis and treatment of breast cancer. Furthermore, researchers used reflexive notes during data collection that were used during data analysis and interpretation to minimize researcher bias.

## Results

This study revealed barriers in screening and treatment of breast cancer that can be divided into three broader categories; individual socio-cultural and structural. Individual barriers encompass lack of awareness of breast cancer, hesitance in accepting social support, and spiritual healing. Feminine sensitivity, stigmatization, and aversion to male doctors were the primary socio-cultural barriers. Additionally, two structural barriers, lack of financial resources and apathetic medical services were identified that inhibit women from diagnosis and treatment of breast cancer**.** Socio-economic and demographic characteristics of study participants are described in Table 1.

1. Individual barriers

This theme captures individual level barriers in diagnosis and treatment of breast cancer. The theme is organized around three sub-themes.

### Lack of awareness

Women in Pakistan hesitate to talk about breast cancer openly because women’s breasts are typically perceived as sexual organs. Women are discouraged to discuss their body in public sphere which inhibits awareness about potential medical issues of woman body. Consequently, knowledge about breast cancer screening is not adequate and the majority of women never had experience of breast self-examinations. Therefore, women are unable to interpret the presence of lumps on their breasts. In this study, most of the participants shared the perspective that women don’t have adequate knowledge about breast cancer, screening and medical treatment. Moreover, many state that they were in a condition of shock when they came to know about their illness. A 47 years old woman expressed her feelings on breast cancer diagnosis:

“Oh! It was a very difficult time in my life. My mind was empty, unable to think of what to do. Poor women like me do not know how and where to get the screening and treatment. No one in my surroundings could guide me about it”. (Age range 44-48, Rural, Married)

Our interviews revealed that most of the patients were in fear when they first found out that they had breast cancer. Moreover, many ended up giving up on the prospects of treatment. They perceived their cancer as incurable and felt that treatment would increase pain and cause medical complications. As a result, many women were afraid to go to the hospital and only visited when cancer had reached an advanced state. This phenomenon is exemplified in the excerpt below:

“When I was told that it is breast cancer, I thought no one can survive after cancer. I started thinking that I am a guest for a few days now. I did not want to go through any medical treatment to waste money of my family when I was going to die anyway. Now, I pray for that doctor who encouraged me for operation and mentioned cases of women who are healthy after removal of the lump”. (Age range 39-43, Rural, Widow)

### Spiritual healing

Religious beliefs are considered as a source of strength and a way to cope with serious diseases. Religious misconceptions, social pressures, and mistaken beliefs contribute to lack of breast cancer screening efforts and delayed help-seeking attitudes among Pakistani women. Participants showed a general lack of awareness about the nature of the disease and its treatment. This led them to alternate treatments which were not always effective. According to a patient:

“When it happened to me, I didn’t know why a lump appeared in my body. I shared it with my sisters and cousins. They shared a few stories of women with the same symptoms and suggested to visit spiritual healers who had reportedly healed those women. I visited those healers for more than 6 months but the lump got more swollen and that scared me further”. (Age range 34-38, Urban, Married)

Females reported that they went for spiritual spells *(Dam-Darood)* and recited some specific verses of Quran^1^ for healing. Some participants stated that they did not know the exact cause of their cancer but felt it was by the will of God.

"I did not know about its (breast cancer) treatment but I had heard about a disease which results in the cutting of breasts. After I got ill, I decided to recite different Surah from the Holy Quran and I drank holy water (water blessed by a spiritual healer) so that I get relief from this painful disease”. (Age range 49-53, Rural, Married)

### Hesitance in accepting social support

It was found that women with breast cancer face lack of social and emotional support from family and other relationships. Due to the perceived insensitive behavior of people, patients avoid meeting friends and neighbors. Patients perceive their social body to be inadequate for public sphere. Consequently, breast cancer patients choose to spend more time in isolation as they do not want to face people. Patients prefer social isolation over available social support to avoid negative body image evaluation of their bodies.

“I face some strange behaviors from my relatives and friends who often avoid me. They think that they will get affected by my disease. Even when some of them want to spend time with me, I simply avoid them because I feel that my relatives stare at my head, eyebrows and chest constantly”. (Age range 39-43, Rural, Married)

The fear of physical deformation, psychological complications and negative anticipations of medical procedures cause suffering to the breast cancer patients. In addition, medicalized view of suffering overlooks the suffering of loss of self during serious illness [27]. Patients don’t want to gain sympathy which makes them feel less valued as a person. They feel loss of self when people put them in a weaker position during social interaction.

Another respondent corroborated with similar gut feelings to maintain the social interaction with family members and relatives.

“I usually get much support from my husband and in-laws. My in-laws take care of my children and they said, do not worry about children. But I avoid meeting those in my family who show sympathy towards me. I need love but I don't need sympathy because it makes me feel doomed”. (Age range 34-38, Urban, Married)

2. Socio-cultural factors

This theme covers social and cultural factors that pose barriers to individual agency of women suffering from breast cancer. Three sub-themes have been discussed under this theme.

### Feminine sensitivity

Women associated breast cancer with a fractured body image and a loss of femininity. This was because they felt that the disease afflicts a body organ that symbolizes femininity and motherhood. Additionally, cancer treatment may lead to skin discoloration, hairlessness, loss of eyebrows and eyelashes, all of which disturb the body image. A participant shared her fear from the treatment;

“One day I came to the hospital for chemotherapy and I was told that my hairs were going to be removed. At that time, I wanted to run away from the hospital. When my hairs were removed I cried a lot. My next fear was that they would cut off my breast. Breasts are the sign of completeness for women. I feel scared when I think about a mastectomy”. (Age range 39-43, Urban, Married)

The breast is a vital organ of the female body. It is a sign of womanliness, sexual beauty, and feelings of being praiseworthy. A mastectomy can damage the woman’s self-image of worthiness and create a feeling of unpleasantness. Some participants described that breast cancer was a disease that threatened their womanhood. Females usually do not opt for the advanced medical treatment of breast cancer to avoid mastectomy. This is illustrated in the account below:

“When I was diagnosed with breast cancer, it challenged my position in my home. My body was being discussed for the first time in my family among both women and men. It made me uncomfortable. Now I have heard that they may operate and cut my breast, I feel very sad and incomplete”. (Age range 24-28, Urban, Single)

### Stigmatization

Participants described that relatives and neighbors started avoiding them after they came to know about their breast cancer. As a result, breast cancer patients faced severe discrimination as their bodies were negatively labeled and viewed as unclean. A woman shared her experience to expose her disease in community:

“I never hid my disease and that was my biggest mistake. Women come to see me but their conversation made me feel guilt and shame. I feel that God gave me this disease because I am a very bad woman. Now I do not go to my husband and children because my in-laws think that they will suffer because of me. I feel pain, sad and cry when some people avoid me and reject me”. (Age range 34-38, Urban, Married)

Participants also reported that they hid their disease initially due to the fear of their husband's rejection. Women held the fear that their husbands could divorce them due to cancer. Breast cancer patients observed that men whose wives had gotten ill had started searching for other females.

“I did not discuss my disease with husband for 6 months because I was confused about how he would react. In our society, some people give an extreme reaction, and, in some cases, husbands can divorce and go for another woman". (Age range 24-28, Urban, Married)

### Aversion to male doctors

Our interviews reported that women were reluctant to visit male doctors for treatment because they did not want to reveal their breasts for screening and treatment to male doctors. Cultural norms and religious values restrain women to get breast cancer treatment and consultation from male physician. Furthermore, husbands and family members also condemn seeking treatment from male physician. Therefore, many women delayed treatment because of the distress caused by discussing a breast-related problem with a male physician. They felt that a pious and good woman should not disclose her body to any male other than her husband.

“When I was directed to a male doctor and he asked me about my disease, it was very embarrassing for me to discuss my breasts with him. It was worse when he asked me to show the tumor. As a Muslim female, I could not imagine that I can ever show my breasts to an unknown male, but when I had no choice, I had to do so”. (Age range 34-38, Urban, Married)

Another respondent expressed;

“Allowing a strange man to look into your body, to talk about and touch your body is the hardest part of this disease. I do not want to even think about these moments” (Age range 39-43, Rural, Widow)

3. Structural barriers

The accounts of breast cancer patients suggest that there are few macro-level institutional factors hindering screening and treatment. Two sub-themes are emerged under this theme.

### Lack of financial resources

The majority of the patients interviewed belonged to the lower socioeconomic background and were unable to bear health care expenditures. Lack of financial resources poses a major barrier to breast cancer treatment. Women do not want to become a burden for their family as cancer treatment is very expensive and usually lower-middle income families are unable to bear these expenses. Consequently, patients delay treatment and hide their pain. This is illustrated in the account below:

“When I came to know about the disease, I thought my treatment expenditures will be a financial burden for my family. I belong to a poor family; if my family spends money over my treatment then they would not have enough for their livelihood”. (Age range 24-28, Rural, Married)

In Pakistan, like many other developing countries, there is a little medical assistance from the government for patients suffering from diseases like cancer. In many cases, most of the cost is borne by the patient and his/her family. So, the patients feel apprehensive while seeking treatment as they think their household income is not enough to spend on medication. Participants described that they are scared of any kind of illness that can cost money and can be stressful for the family.

“I tried to ignore the breast lump when I observed it in my body. I was reluctant to go for treatment because my family was not financially stable and they could not afford my treatment expenses. When the pain was out of my control, my children forced me to visit the doctor”. (Age range 24-28, Urban, Divorced)

Another woman pointed out that:

“While starting my treatment I faced severe financial problem. We are poor people and we have not enough money to spend on such expensive treatment. I often think that instead of providing bread to my children, I am spending money on my treatment. I came to hospital, when I thought that if I die, who will look after them (children), and who will take household responsibilities….?” (Age range 24-28, Rural, Divorced)

In Pakistan, health insurance is not common and most of women remain economically dependent on their husbands. Moreover, they are conscious about meeting the needs of their children and other family members and often neglect their own health.

### Apathetic medical services

A majority of cancer patients are unable to receive treatment facilities and even pain management in case of non-curable cancers. In public hospitals, majority of cancer patients’ treatment is being refused due to the limited capacity of in-patients and the unavailability of oncologists. Moreover, most of the primary health care centers/hospitals do not have cancer screening facilities. The hospitals that have cancer treatment facilities are unable to provide satisfactory services to patients. A participant shared her experience:

“Initially, I faced a terrible experience when I visited a renowned hospital. The hospital administration refused to provide in-patient care because they felt I was too old. They made me feel like a burden and I realized that old people are not needed by society. I visited the hospital for treatment and their (physicians’) attitude made my sickness worst.” (Age range 44-48, Urban, Married)

In contrast to the above findings, a few patients shared that the doctors' attitude was not a barrier, but lack of infrastructure is the major problems for patients. Participants in the study had positive remarks about doctors but were unhappy with other paramedical staff and hospital administration.

“I was angry when the doctor was absent, and I was forced to wait for more than 3 hours even though I had an appointment. Sometimes the behavior of staff also discouraged me, and I thought that I should quit treatment. The ward boys and nurses responded very harshly whenever I made any request.” (Age range 44-48, Urban, Married)

## Discussion

The interviews explored the multifaceted barriers to diagnosis and treatment of breast cancer in Pakistan. Interviews reported that women hesitate to expose breast cancer at early stage and typically perceive breast as a sexual organ. Knowledge about breast cancer screening is optimal and women did not have knowledge of breast screenings, self-examination, treatment facilities when they first observed lump in their bodies. Different studies from Muslim majority countries have also reported that lack of awareness of the disease causes delay in the screening and treatment of breast cancer [28–31].

Lack of knowledge about appropriate breast cancer screening and treatment can cause negative perceptions and their unrealistic reliance on spiritual and traditional treatment. It may lead women to adopt spiritual and traditional healing. Furthermore, in societies where people follow religious beliefs, they often associate calamities and afflictions with the will of God and believe that supernatural interventions can cure the disease. In this study, some of the participants reported that they were unaware of the causes of their disease and felt that only God could heal them. This response is similar to reports from both African American and Asian American women who believed that their future was in God's hands as God has more control over the development of cancer [32, 33]. Consequently, consulting spiritual and traditional healers may result into treatment delay [34].

To cope with illness related distress and stigma, patients utilize social support of family and friends. Studies have demonstrated that breast cancer patients are not provided with appropriate social support due to older age, the severity of cancer treatment, and co-morbidities [35, 36]. A plethora of literature suggests that patients with serious illness need social and emotional support [37]. In contrast, findings of the present study suggest that patients evaded available social support from family members and friends. A few participants reported that they avoided receiving available social support because of providers’ gazes which negatively impact patient’s self-esteem. This finding is unique which demands further exploration. Participants also avoid receiving social support due to possible sympathy. Studies suggest that stigma reproduction and maintenance in community silence patients push them to social isolation [38] which further deteriorates their psychological health [39]. Theoretical foundation of stigma can be found in work of Ervin Goffman who referred it as symbols cut or burnt onto the body to denote moral status [40]. Rosman found that stigma causes disruption to interaction with ‘normals’ among cancer patients [41].

Interviews also revealed that women had physical and emotional concerns for the treatment of breast cancer. Participants were fearful of treatment side effects such as: hairlessness and the loss of eyebrows and eyelashes. They felt that these changes would disfigure their body image. Respondents also reported that they purposely delayed their treatment due to the fear of mastectomy and loss of femininity through seeking advance medical treatment. Studies from different women populations in Asia, Africa, Europe, and America have also reported that women are highly concerned about their breasts, as they believe breasts define their identity and acceptance in society [42–45]. Breast cancer treatments often result in serious changes in the appearance of patients, including breast asymmetry and changes in skin texture and sensitivity [46]. Interviews also report that appearance-related issues resulting from breast cancer treatment were a major source of disruption of the women’s sense of self which affected their self-esteem and social functioning. Women with breast cancer go through a transition of changed body image after the diagnosis and surgery [47]. They need social and emotional support to deal with this renegotiation of their body identity. Negative body image evaluation is one of the psychological challenges breast cancer patients face [48]. Cash’s cognitive-behavioral model of body image explains that interpersonal experiences greatly influence one’s body image [49].

Furthermore, interviews reported that women did not disclose their disease to avoid social stigma and were reluctant for screening. Breast cancer is considered a contagious disease by family members, most of whom choose to avoid breast cancer patients. Studies from Pakistan, south Korea, and the USA reported that women face a plethora of emotional problems after diagnosis including: isolation, employment issues, stress, blame of bad luck, and social critique [32, 50, 51]. In addition, the fear that breast cancer reflects upon the bad character of women, shyness, and fear of husband's separation also contributed to hiding and delayed in breast cancer screening in India, Iran and Asian American women in the USA [52–54].

Interviews with breast cancer patients pointed out that they delay their medical treatment because of distress in discussing the breast-related problems in front of a male physician. In south Asian countries women are usually reluctant discussing breast related problems [55]. Pakistani women are brought up in a culture where breasts are considered as sensitive parts of a woman’s body which have to be concealed all the time. Such socialization inhabits women to expose their breast to male physicians. A study from Pakistan also reported that women feel unprotected to let male practitioners do their breast examinations [22]. Similarly, different studies from south Asian countries mentioned that women desire modesty and respect for their privacy and that is the major barrier for screening and treatment of breast cancer [39–41].

The other major barrier that was repeatedly emerged from the interviews of women was lack of financial resources to seek screening, diagnosis and treatment of breast cancer. In Pakistan cancer screening and treatment facilities are scarce and expensive and patients usually avoid seeking care due to lack of money. Moreover, patriarchal social structure of society provides lesser economic opportunities to women making them dependent on male breadwinners. Studies from Pakistan also reported that poor economic conditions pose an important barriers to breast cancer screening and treatment [12, 23]. In such a situation, patients prefer to seek alternative treatment such as to visit the shrines or religious leaders (faith healers), and traditional healers instead of attending hospitals. In traditional societies it has also been found that women initially seek spiritual treatment until their tumor starts growing [50]. Furthermore, women who belong to lower socioeconomic class mostly avoid attending hospitals due to prohibitive distances, travel costs, and treatment expenses and use holy water and Taweez (amulet) to cure the disease. Delay of screening and diagnosis of breast cancer due to lack of money insurance and transportation cost is cited in many studies across developed and developing countries [50, 52, 53, 58].

## Conclusions and implications

This study has identified multifaceted barriers to breast cancer diagnosis and treatment including individual, socio-cultural and structural factors. Individual barriers encompass a lack of awareness, spiritual healing, and hesitance in accepting social support. Whereas feminine sensitivity, stigmatization, and aversion to male doctors were found to be the socio-cultural factors that inhibit women from screening and treatment of breast cancer. In addition, lack of financial resources and apathetic medical system were structural barriers for women. Implementing a threefold strategy to deal with psychological, socio-cultural, and structural barriers in the way of breast cancer screening and treatment can be fruitful. Pakistan is a resource-constrained country with significant women-illiteracy that creates deplorable situation for women suffering from breast cancer. Thus, serious educational interventions are needed to raise awareness about breast cancer. On a community level, breast health programs should be introduced so that the death rate of cancer can be minimized. Policymakers, healthcare providers and researchers should take culturally informed measures to improve the diagnosis and treatment procedure in Pakistan.

## Limitations of study

The current study has two principal limitations. The sample of this study was comprised of women from Punjab which is a large province with diverse sociocultural settings making it difficult to reflect the findings at border level. Furthermore, data were collected from only one hospital that may have potential bias from the participant’s views.

## Supplementary information

**Additional file 1**. Semi-structured interview guide.

## Data Availability

The datasets used and/or analyzed during the current study are available from the corresponding author on reasonable request.

## Abbreviations

PINUM: Punjab Institute of Nuclear Medicine
GCUF: Government College University Faisalabad
ARB: Advance Research Board
USA: United States of America

## Footnotes

1 Quran is the central religious book of Islam, which Muslims believe to be a revelation from God.
