# Supplementary material for: Fears and barriers: problems in breast cancer diagnosis and treatment in Pakistan
Source: BMC Womens Health. 2021 Apr 14;21:151. doi: 10.1186/s12905-021-01293-6 (PMC8045297; doi:10.1186/s12905-021-01293-6)
Supplement: Supplementary file 1 — Additional file 1. Semi-structured interview guide. [file 12905_2021_1293_MOESM1_ESM.docx]

Fears and Barriers: Problems in Breast Cancer Diagnosis and Treatment in Pakistan

**Interview guide:**

- What is your age?
- How many children do you have?
- How much formal education you have attained?
- I would like to know about your marital status?
- Can you tell me about your living area?
- What is your household monthly income?
- How many years ago you were diagnosed with breast cancer?
  - Explore the process how she got know about this disease?
  - Explore the process of diagnosis of breast cancer.
  - Explore the health seeking behavior of patients.
- Please explain your experience of discovery and reaction to disease?
- What are the societal barriers that prevent women from participating in breast cancer screening?
  - *Was there any barrier caused by family?*
  - *Any trouble caused by hospital?*
  - *Anything at hospital that discourages patients to visit hospital?*
- Please tell me the problems of seeking treatment of breast cancer?
  - Probe on socio-cultural barriers
  - Lack of resources
  - Exploring major barriers for seeking treatment
  - Economic constraints and seeking care
- How this disease has affected your life?
  - *What particular changes it has created in your life?*
  - *Disease’s impact on your behavior?*
  - *Family’s response? What was the reaction of your family and what was level social support you received?*
- How was family’s social support during this experience?
  - *Was provided social support enough during your illness?*
